# Elevated Serum Gamma-Glutamyltransferase (GGT) Activity and the Development of Chronic Kidney Disease (CKD) in Cigarette Smokers

**DOI:** 10.5812/numonthly.13652

**Published:** 2013-11-13

**Authors:** Yuka Noborisaka, Masao Ishizaki, Michiko Yamazaki, Ryumon Honda, Yuichi Yamada

**Affiliations:** 1 Department of Social and Environmental Medicine, Kanazawa Medical University School of Medicine, Ishikawa, Japan

**Keywords:** Smoking, Gamma-glutamyltransferase, Kidney Diseases, Proteinuria, Glomerular Filtration Rate

## Abstract

**Background:**

Elevated serum gamma-glutamyltransferase (GGT) is predictive of various cardiovascular (CV) risk factors including chronic kidney disease (CKD). Elevated serum GGT has been recognized in smokers who are likely to develop CKD, but no study has focused on serum GGT and CKD in smokers.

**Objectives:**

The aim of this study was to clarify the associations between cigarette consumption, elevation of serum GGT and the development of proteinuria and CKD.

**Patients and Methods:**

A retrospective 6-year observational study was conducted on 2,603 male workers aged between 40 and 64 years. Incidences of proteinuria detected by dipstick and CKD defined by proteinuria and/or reduced estimated glomerular filtration rate (eGFR) measured in health check-ups were determined 6 years later for those who had been free of them at baseline.

**Results:**

Higher means of serum GGT in smokers than in nonsmokers at baseline, and a higher incidence of elevated serum GGT in smokers than in nonsmokers during the 6-year period were recognized only for alcohol consumers. Incidences of proteinuria and moderate or severe CKD which has a high risk of future renal failure or CV disease were higher in the subjects with greater cigarette consumption or a higher serum GGT level. Multiple logistic regression analyses adjusting for major CV risk factors showed a significant interactive effect between smoking and elevated serum GGT on the development of proteinuria and an additive effect of smoking and serum GGT on the development of high-risk CKD.

**Conclusions:**

Elevation of serum GGT in smokers, to a large extent, depends on the associated alcohol consumption. Elevated GGT in smokers plays at least a partial role in the development of CKD, mainly proteinuria, and the underlying mechanisms remain to be elucidated.

## 1. Background

Elevated serum gamma-glutamyltransferase (GGT) activity, a well-known biological marker of excessive alcohol consumption or liver injury including fatty degeneration in obese persons, has recently been demonstrated to predict the development of cardiovascular (CV) risk factors such as hypertension (1-5), diabetes mellitus (DM) (6-11), the metabolic syndrome (12-14), and even cardiovascular disease (CVD) itself (11, 15-18). These findings suggest the possible relevance of elevated serum GGT activity in the development of chronic kidney disease (CKD) because these CV risk factors are also known to contribute to the development of CKD, but the literature on this issue has been controversial and very limited in number (19-22).

As well as hypertension, DM and obesity, chronic cigarette smoking is a major risk factor for the development of CKD in the general population although the exact biological mechanisms underlying the link between smoking and CKD remain unclear (23). Elevation of serum GGT activity has been recognized even in cigarette smokers (24-28).

## 2. Objectives

None of the previous studies, however, have focused on the relevance of elevated serum GGT activity in smokers with regard to the development of CKD, and this prompted us to clarify the association by means of a retrospective 6-year longitudinal observation of Japanese male workers.

## 3. Patients and Methods

Details of the characteristics of the study subjects were described in our previous report (29). Originally there were 2,712 men aged between 40 and 64 years who had received annual health check-ups at workplaces including the measurement of serum creatinine (Cr) concentration and thus estimated glomerular filtration rate (eGFR) during both 2003 and 2009, performed by an occupational health service organization. Further, exclusion of 109 men because of the lack of critical data, mainly body weight or urinalyses, resulted in 2,603 men being enrolled as the final subjects.

In the health check-ups, body height (m) and weight (kg) were measured with jacket and shoes removed, and 0.5 kg was subtracted during spring to summer and 1.0 kg during autumn to winter for the net body weight. Body mass index (BMI) was calculated as the net body weight divided by the height squared. Blood pressure (mmHg) was measured using an automatic manometer in the sitting position with a cuff maintained at heart level after resting on a chair for five minutes or longer. Urinary protein was detected in the spot samples collected in the morning with a dipstick and defined semi-quantitatively. From age (year) and serum Cr concentration (mg/dL) measured enzymatically, eGFR (mL/min/1.73 m^2^) was calculated using the equation proposed by the Japanese society of nephrology (JSN) (30). Concentrations of total cholesterol (Tchol), triglycerides (TG), HDL-cholesterol (HDLc), LDL-cholesterol (LDLc), and uric acid (UA) in serum were measured in the fasting venous blood, as well as serum hepatic enzymes activities including GGT using an automatic analyzer. At the same time, the concentration of fasting plasma glucose (FPG) and hemoglobin A1c (HbA1c) were measured using automatic analyzers.

Hypertension was defined as being present when the subject was being treated with antihypertensive agents and/or when the blood pressure measured in the health check-ups was 140/90 mmHg or higher. DM was considered present when the subject was being treated with hypoglycemic agents and/or had a FPG of 126 mg/dL or HbA1c of 6.5% (NGSP) or higher. Subjects were defined to have dyslipidemia such as hypercholesterolemia when they showed a Tchol of 220 mg/dL or a LDLc of 140 mg/dL or above, hypertriglyceridemia when they showed a TG of 150 mg/dL or above, and low HDL-cholesterolemia when they showed a HDLc of less than 40 mg/dL. Use of medications for dyslipidemia was not considered in the definitions because of the vagueness of the subjects’ answers. Hyperuricemia was defined as present when the subject was being treated with medicines and/or had a serum UA of 7.5 mg/dL or higher. Serum GGT activity in the subjects was divided into 3 categories of less than 40 U/L (the upper 95% value in non-obese male non-drinkers), 40~89 U/L, and 90 U/L (the upper 95% value in generally healthy men) or higher which was defined as elevated serum GGT. Smoking habit was classified into non-smokers including ex-smokers, smokers consuming up to 1 pack of cigarettes per day and those consuming more. For alcohol consumption, the subjects were classified into nondrinkers, drinkers consuming up to 59 mL of ethanol per day, and those consuming 60 mL per day or more and subjects were scored from 0 to 2 for statistical analyses.

The levels of eGFR (G) were classified into G1: 90 mL/min/1.73 m^2^ or higher, G2: 60-89.9, G3a: 45-59.9, G3b: 30-44.9, G4: 15-29.9, and G5: less than 15.0. Although the amount of 24 h albumin excretion in urine (A) is required in the criteria of CKD severity proposed by KDIGO (31), it could not be determined by a dipstick measurement in spot urine samples in the health check-ups. In the present study, therefore, the dipstick proteinuria (-/±) was tentatively assumed to accord with normal (A1), dipstick (1+) with mild proteinuria (A2), and dipstick (2+ or above) with marked proteinuria (A3) (32). The subjects were then defined as being free of CKD when they had eGFR of G1 or G2 without proteinuria (A1). The subjects were defined to have mild CKD when eGFR was G1 or G2 with mild proteinuria (A2) or eGFR was G3a without proteinuria (A1), and defined to have moderate CKD when their eGFR was G1 or G2 with marked proteinuria (A3) or eGFR of G3a with mild proteinuria (A2) or eGFR was G3b without proteinuria (A1). The subjects were defined to have severe CKD when they had eGFR of G3a with marked proteinuria (A3), eGFR of G3b with proteinuria (A2 or 3), or eGFR of G4 or G5 regardless of proteinuria. The cause of kidney damage (C) could not be determined in the present study. Moderate and severe CKD were defined as high-risk CKD in the present study because of the high future risk of end stage kidney disease (ESKD) and CVD associated with these levels of renal dysfunction (31).

The incidences of proteinuria and total CKD comprising any degree of severity in 2009 were determined in the subjects who were free of all CKD signs at baseline in 2003, and the incidence of high-risk CKD was determined in the subjects including those having mild CKD with mild proteinuria or reduced eGFR of G3a in 2003 according to the 3 x 3 categories of cigarette smoking and serum GGT activity. Next, multiple logistic regression analysis was conducted to test the significance of the contributions of smoking and serum GGT to the development of proteinuria, total CKD and high-risk CKD, first in a model including only current smokers but not elevated serum GGT as an independent variable, second in a model including both smoking and elevated serum GGT as additive variables, and finally in a model including the two variables with an interactive effect, adjusting for major CV risk factors in the analyses on proteinuria and total CKD, and adjusting for preceding proteinuria and reduced eGFR as well as the CV risk factors in the analyses on high-risk CKD.

Written informed consent was obtained from all the subjects by the health service organization regarding the use of data collected in the health check-ups for academic purpose in anonymous forms, and the present study was designed to analyze the data anonymously in an unlinkable fashion that was provided by the health service organization and approved by the ethics committee of Kanazawa Medical University.

## 4. Results

**Figure 1.**
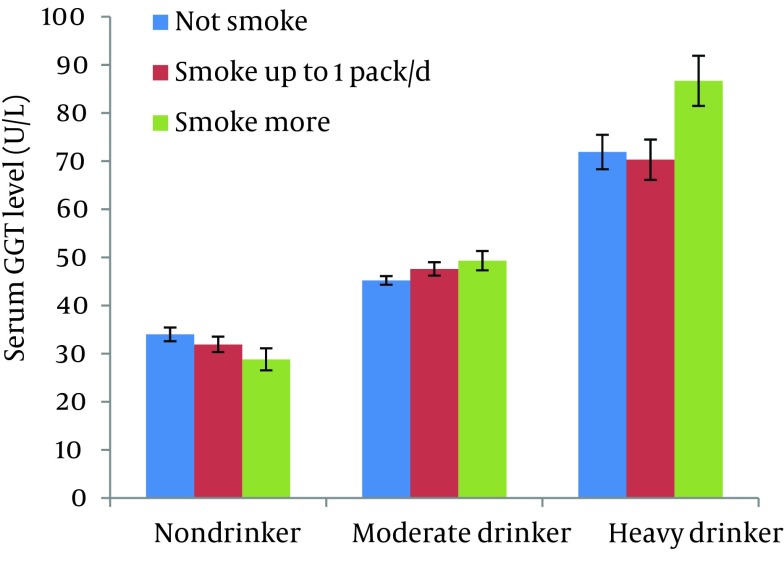
Geometric Means and Standard Errors (Upper Bars) of Serum Gamma-glutamyltransferase (GGT) Activity at Baseline in Middle-aged Men According to Alcohol and Cigarette Consumption Being Adjusted for Age and Body Mass Index

**Figure 2.**
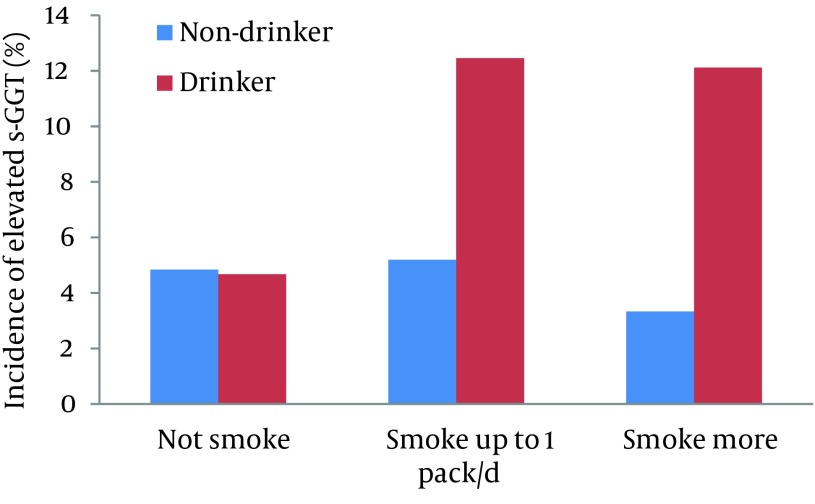
Incidence of Serum Gamm-glutamyltranseferase (GGT) Activity of 90 U/L or Higher in Middle-aged Men According to Alcohol and Cigarette Consumption During a 6-year Period

Geometric means and standard errors (shown as upper bars) of serum GGT activity at baseline of 2003 in the subjects divided by the categories of alcohol and cigarette consumption adjusted for age and BMI are illustrated in Figure 1. Serum GGT activity seemed lower in smokers than in nonsmokers in the subjects who did not consume alcohol while it tended increased with increases in the number of cigarettes consumed by alcohol consumers. The direct effect of alcohol consumption and the interactive effect between alcohol and cigarette consumption on serum GGT level were statistically significant (P = 0.001 and 0.03, respectively) while the direct effect of smoking was not significant (P = 0.836). Further, the incidence of elevated serum GGT of 90 U/L or higher during the 6-year period in the subjects who did not show it at baseline was 136 (6.38%) in 2132 men. As illustrated in Figure 2, the incidence was conspicuously higher for cigarette smokers than for non-smokers, but was found only in those consuming alcohol. Statistical analysis showed that the interactive effect between alcohol and cigarette consumption on the incidence of elevated serum GGT was significant (P = 0.013) while the direct effects of alcohol and cigarette consumption were not significant (P = 0.897 and 0.635, respectively). 

**Figure 3.**
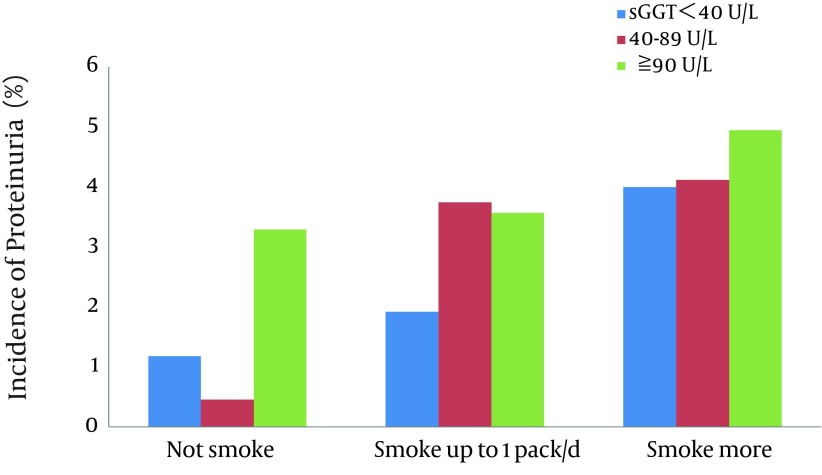
Incidence of Proteinuria in Middle-aged Men According to Cigarette Consumption and Serum Gamma-glutamyltransferase (GGT) Levels During a 6-year Period

**Figure 4.**
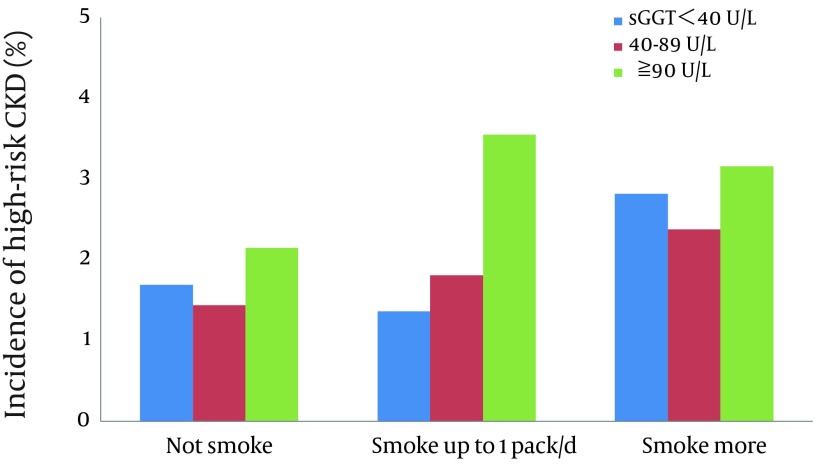
Incidence of High-risk CKD in Middle-aged Men According to Cigarette Consumption and Serum Gamma-glutamyltransferase (GGT) Levels During a 6-year Period

The numbers of subjects with proteinuria and total CKD during the 6-year period between 2003 and 2009 were 55 (2.25%) and 404 (16.5%) in 2445 men, respectively, while that of high-risk CKD was 50 (1.93%) in 2584 men. The incidence of proteinuria in the subjects divided by the categories of cigarette consumption and serum GGT activity is illustrated in Figure 3. The incidence of proteinuria was generally increased with either increases in the number of cigarettes consumed or elevations of serum GGT. The appearance of proteinuria was especially high in those with elevated serum GGT irrespective of smoking and in heavy smokers irrespective of serum GGT levels. Figure 4 illustrated the incidence of high-risk CKD in the subjects. It was also generally increased with increases either in the number of cigarettes consumed or elevations of serum GGT. 

Independent effects of current smoking and elevated serum GGT on the development of proteinuria, total CKD and high-risk CKD were tested by multiple logistic regression analyses adjusting for major CV risk factors such as age, BMI, alcohol consumption, hypertension, DM, hypercholesterolemia, hypertriglyceridemia and low HDL-cholesterolemia, and hyperuricemia. Mild proteinuria and mildly reduced eGFR were also adjusted for in the analysis of the development of high-risk CKD. The results are summarized in Table 1. The effects of current smoking on the development of proteinuria and high-risk CKD were both strongly significant, and were not replaced by the effects of elevated serum GGT as shown in model 2. As shown in model 3, the interactive effect between smoking and serum GGT significantly influenced the appearance of proteinuria although it reduced the magnitude of the effect of both current smoking and elevated serum GGT. For the incidence of high-risk CKD, the effects of current smoking and elevated serum GGT seemed simply additive since the interactive effect was not significant. Neither smoking nor elevated serum GGT was significantly related to the development of total CKD. 

**Table 1.** Results of the Multiple Logistic Regression Analyses of the Contributions of Current Smoking, Elevated Serum Gamma-glutamyltransferase (GGT) Activity and the Interactive Effect Between Smoking and Serum GGT on the Development of Proteinuria, Total and High-risk CKD During a 6-year Period in Middle-aged Men.

Models	Proteinuria^a^			Total CKD^a^			High-risk CKD^a^		
	OR	(95% C.I.)	P	OR	(95% C.I.)	P	OR	(95% C.I.)	P
**1^b^**									
Current smoking	3.23	(1.72-6.06)	< 0.001	0.93	(0.75-1.17)	0.541	2.23	(1.17-4.27)	0.015
**2** ^**c**^									
Current smoking	3.18	(1.69-5.97)	< 0.001	0.93	(0.75-1.17)	0.548	2.21	(1.15-4.23)	0.017
Elevated serum GGT	1.35	(0.68-2.67)	0.387	0.91	(0.67-1.24)	0.553	2.43	(1.14-5.20)	0.022
**3** ^**d**^									
Current smoking	5.57	(2.40-12.9)	< 0.001	0.94	(0.74-1.21)	0.646	2.32	(1.08-4.97)	0.031
Elevated serum GGT	4.36	(1.44-13.2)	0.009	0.94	(0.62-1.42)	0.764	2.68	(0.89-8.02)	0.078
Smoking x GGT	0.18	(0.05-0.70)	0.013	0.94	(0.53-1.66)	0.839	0.85	(0.22-3.24)	0.810

^a^ For the definitions, refer to text.

^b^ The model only included current smoking as an independent variable as well as major confounding variables which included mild proteinuria and mildly reduced GFR in the analysis of high- risk CKD.

^c^ The model included smoking and elevated serum GGT as additive variables.

^d^ The model included smoking, elevated serum GGT and the interactive effect between smoking and elevated serum GGT.

## 5. Discussion

In some previous studies demonstrating an elevation of serum GGT in smokers, this elevation was noted to be independent of alcohol consumption (25, 26), whereas others showed an interactive effect between alcohol and cigarette consumption (24, 27, 28). The present findings are consistent with the latter, namely elevations of serum GGT related to smoking were clearly observed only in subjects consuming alcohol but not in those not in both the cross-sectional and longitudinal observations.

The reason for this discrepancy in the studies is not clear, but some bias related to subject selection may have affected the present results. Elevation of serum GGT in non-alcohol consumers is likely to be most commonly attributable to obesity, another major cause of serum GGT elevation in the general population (33). Obese smokers having a high serum GGT may have been more vulnerable to various diseases making them more likely to drop out during the observation period, which might have accounted for the lower serum GGT levels in smokers without alcohol consumption and incidence of their elevated serum GGT has been underscored. Even considering the effects of the selection bias, the present findings suggest that elevation of serum GGT in smokers may depend to a great extent on the associated alcohol consumption.

The biological mechanisms underlying the elevation of serum GGT due to cigarette smoking have not been elucidated. Some ingredients of tobacco leaf or smoke such as nicotine or benzene may induce the enzyme or may injure the liver cells (28, 34). However, the interactive effect between alcohol and cigarette consumption on the elevation of serum GGT observed in the present study supports the contention that smoking would mainly enhance the inflammatory process in the liver that was initiated by alcohol consumption (27).

As shown in Figure 3 and 4, increases in cigarette consumption and elevations of serum GGT both seemed to relate to the development of proteinuria and high-risk CKD. The development of total CKD comprising any degree of severity was, however, not related to either cigarette consumption or serum GGT and thus not illustrated. Multiple logistic regression analyses adjusting for major CV risk factors showed that current smoking exerts a strong effect on the development of both proteinuria and high-risk CKD, and that elevated serum GGT showed a significant effect in an interactive way with smoking on the development of proteinuria and in an additive way on the development of high-risk CKD. The results suggest that elevated serum GGT alone does not account for the entire biological link of smoking with CKD, but plays at least a partial role in the development of CKD in smokers, especially proteinuria and thus high-risk CKD that was often accompanied with proteinuria. 

On the other hand, neither smoking nor elevated serum GGT was related to the development of total CKD, most cases of which were characterized by a mildly reduced GFR without proteinuria. These findings could explain the controversial report of Teppala et al. (21) showing no significant association between elevated serum GGT and the prevalence of CKD defined as reduced eGFR of less than 60 mL/min/1.73 m^2^ in the participants of the National Health and Nutrition Examination Study (NHANES) 1999-2002, versus that of Targher et al. (20) showing a significant association with the prevalence of CKD defined as reduced eGFR and/or abnormal albuminuria in the same study program of NHANES 2001-2006, although both studies had a major limitation as cross-sectional observations.

The biological link between elevated serum GGT and the development of major CV risk factors and disease has been variously proposed. Dyslipidemia (35) or hyperinsulinemia due to increased insulin resistance (12, 36, 37) often associated with elevated serum GGT may relate to the development of these risk factors. More recent epidemiological studies have shown the associations of elevated serum GGT with inflammatory or oxidative processes (27, 38-41) which have been speculated to underlie the progression of atherosclerosis and CVD. Basic studies have demonstrated that GGT plays an important role in the regulation of glutathione, a representative defense mechanism against oxidative stress (42). Such inflammatory and oxidative processes may be implicated in the association of elevated serum GGT with endothelial dysfunction (22) cumulating in the development of proteinuria. Alcoholic liver injury has been shown to induce oxidative stress (43), and thus smoking may enhance oxidative stress and inflammation in the liver, causing further elevation of serum GGT and development of proteinuria although further investigations are required to elucidate the exact underlying mechanisms.

A possible selection bias of the subjects and the well-recognized weak reliability of dipstick measurements for proteinuria and estimated GFR are the major limitations of the present study. More reliable methods such as albumin-creatinine ratio in urine and the actually measured GFR are required to confirm the present findings. In addition, the present study was conducted only for male workers. In female workers, the numbers of alcohol and cigarette consumers were very small, and no significant association was observed between cigarette consumption and elevations of serum GGT. Larger scale studies may reveal the association of elevated serum GGT and CKD even in female smokers. The present findings can be summarized as follows; elevated serum GGT was detected in smokers, but it greatly depended on the associated alcohol consumption. Elevated serum GGT alone did not explain the entire effect of cigarette smoking on the development of CKD, but appeared to play at least a partial role in the development of CKD, especially proteinuria, in smokers.
